# A unique association of arrhythmogenic right ventricular dysplasia and acute myocarditis, as assessed by cardiac MRI: a case report

**DOI:** 10.1186/s12872-016-0412-2

**Published:** 2016-11-21

**Authors:** Andrea Ponsiglione, Marta Puglia, Carmine Morisco, Luigi Barbuto, Antonio Rapacciuolo, Mario Santoro, Letizia Spinelli, Bruno Trimarco, Alberto Cuocolo, Massimo Imbriaco

**Affiliations:** Department of Advanced Biomedical Sciences, University “Federico II”, Via Pansini, 5, 80123 Naples, Italy

**Keywords:** Myocarditis, Cardiac, Magnetic resonance imaging, Right ventricle, Case report

## Abstract

**Background:**

Arrhythmogenic right ventricular dysplasia (ARVD), is a genetic disorder of the heart, which mainly involves the right ventricle. It is characterized by hypokinetic areas at the free wall of the right ventricle (RV) or both ventricles, where myocardium is replaced by fibrous or fatty tissue. ARVD is an important cause of ventricular arrhythmias in children and young adults. Although the transmission of the disease is based on hereditary, in young adults it may not show any symptoms. The main differential diagnoses with other frequent etiological causes of sudden arrhythmia are: idiopathic outflow tract ventricular tachycardia of the RV, myocarditis, dilated cardiomyopathy and sarcoidosis.

**Case presentation:**

We describe an unusual case of a 44-year-old woman who was hospitalized for ventricular tachycardia, deep asthenia and dyspnoea with no previous history of cardiac disease. The patient had a ten-year history of palpitations, which started immediately after her last pregnancy. She was diagnosed with both acute/subacute viral myocarditis and arrhythmogenic right ventricular dysplasia, based on established clinical and cardiac MRI criteria. After the diagnosis the patient received an automatic implantable cardioverter defibrillator. Currently, she is on clinical follow-up with no apparent further complications.

**Conclusion:**

Analyzing this rare case, we have shown the link between myocarditis and arrhythmogenic right ventricular dysplasia, and how important is to perform a cardiac MRI, in the context of acute myocarditis and ventricular arrhythmia.

## Background

Arrhythmogenic right ventricular dysplasia (ARVD), is a genetic disorder of the heart, that mainly involves the right ventricle [1, 2]. It is characterized by hypokinetic areas at the free wall of the right ventricle (RV) or both ventricles, where myocardium is replaced by fibrous or fatty tissue. ARVD is an important cause of ventricular arrhythmias in children and young adults. Although the transmission of the disease is based on hereditary, in young adults it may not show any symptoms. The main differential diagnoses with other frequent etiological causes of sudden arrhythmia are: idiopathic outflow tract ventricular tachycardia of the RV, myocarditis [3], dilated cardiomyopathy and sarcoidosis. We hereby describe a rare case of a patient with both acute viral myocarditis and ARVD, as assessed by echocardiography and cardiac MR (CMR).

## Case presentation

**Fig. 1:**
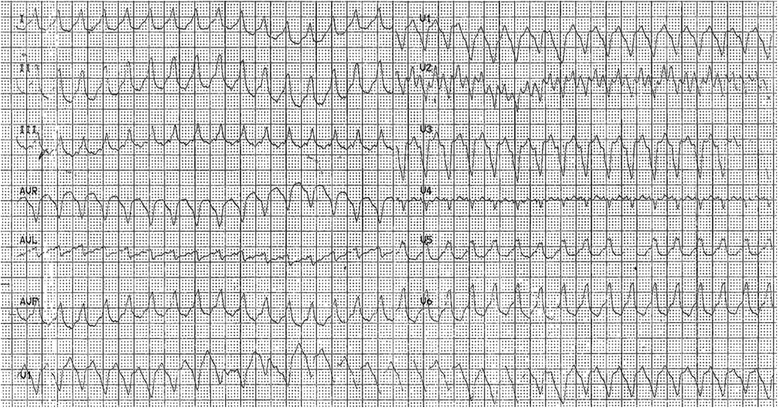
ECG performed on patient admission, showing a ventricular tachycardia with heart rate of 192 bpm

The patient is a 44 year-old woman with a ten-year history of palpitations, who started immediately after her last pregnancy. She wasn’t an active smoker and had a family history of cardiac disease (her father died at the age of 49 years-old for myocardial infarction). The patient was admitted to the emergency department for palpitations, deep asthenia and dyspnoea appearing suddenly when she was at home, under resting conditions. The patient was not febrile and did not show any episodes of chest pain or syncope. At physical examination, the patient revealed no symptoms of right heart failure. ECG performed at her admission showed a ventricular tachycardia (VT) with heart rate of 192 bpm (Fig. 1). Initially the VT was treated by intravenous bolus of lidocaine. For the resistance of the VT to lidocaine, was injected an intravenous bolus of amiodarone and adenosine. In response to the combined antiarrhythmic treatment, the patient had a drop of systemic blood pressure (80/50 mmHg), developed bradycardia (junctional rhythm at 38 bpm) associated with dizziness. For this reason she was moved to the coronary care unit (CCU). Here she was treated with temporary endocardial pacing and hydration therapy. Within 24 h the patient restored the sinus rhythm and normal blood pressure. ECG recorded in sinus rhythm, showed frequent polymorphic ectopic ventricular beats associated with negative T waves in right precordial leads. On admission in CCU the blood tests revealed an elevated troponin level (23 ng/mL, normal value: <5 ng/mL ng/mL) and an elevated CK-MB level (1240 IU/L, normal value < 120 IU/L). The inflammation markers were also elevated with a C-reactive protein (CRP) of 30,5 mg/L (normal value: < 8 mg/L) and 21.000/mL white blood cells (normal value: 4000-10000/mL). Her plasma electrolyte levels were normal.

**Fig. 2:**
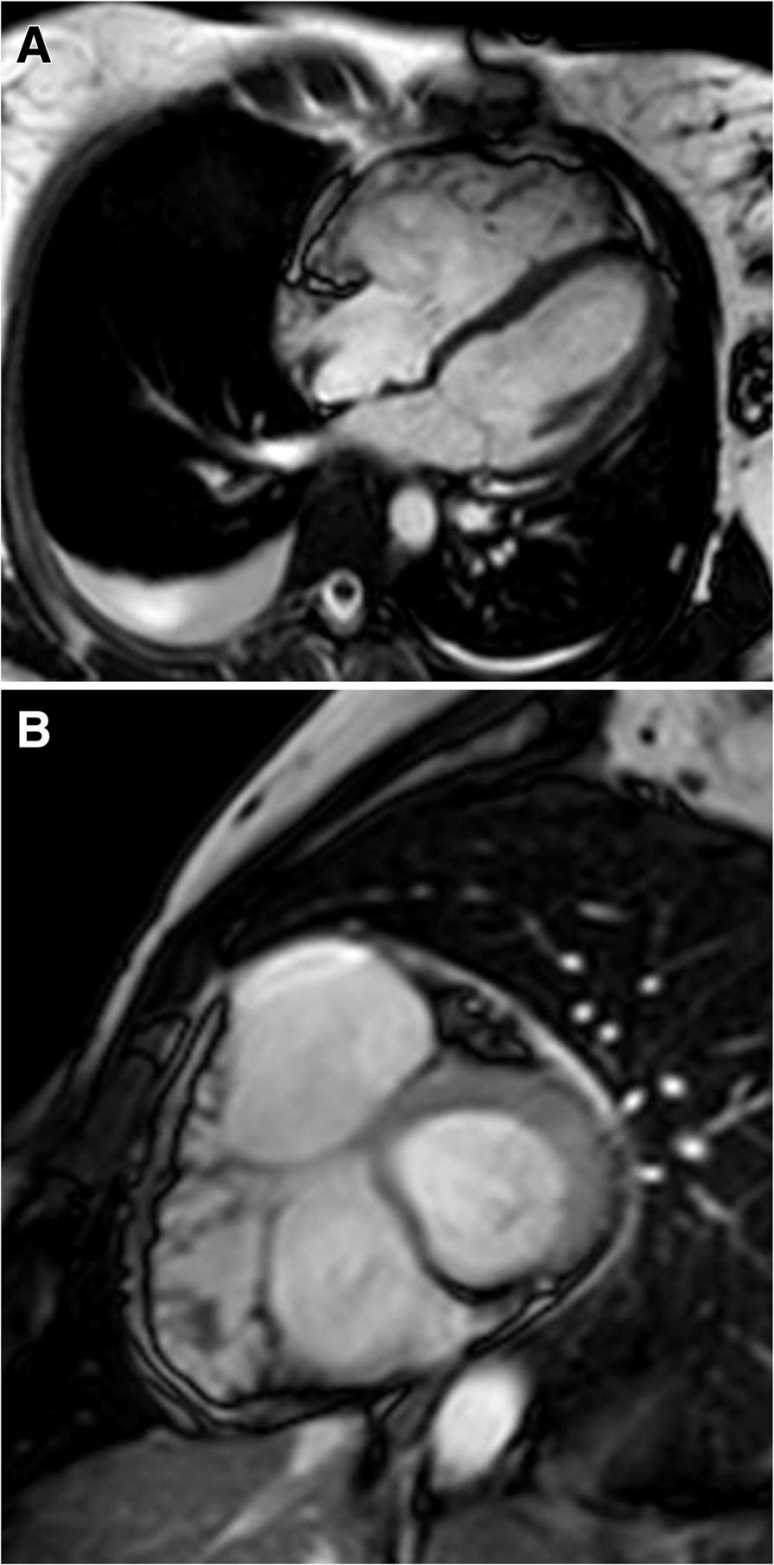
Horizontal long axis (**a**) and short axis (**b**) balanced FFE images showing a dilated right ventricle, with diffuse free wall iper-trabeculation and dyskinesia, and mid-RV free wall pseudo-aneurysm. Bilateral pleural effusion is also present; more conspicuous on the right side

**Fig. 3:**
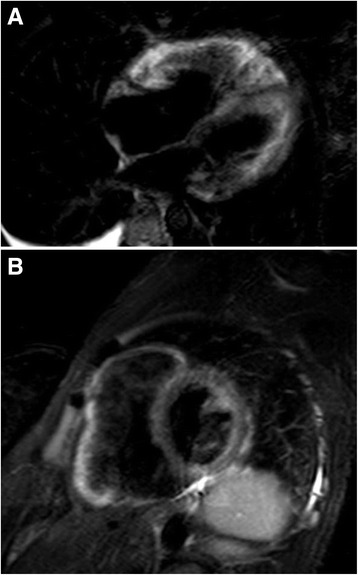
Horizontal long axis (**a**) and short axis (**b**) STIR-T2W images showing a diffuse edema of the right ventricular free wall

**Fig. 4:**
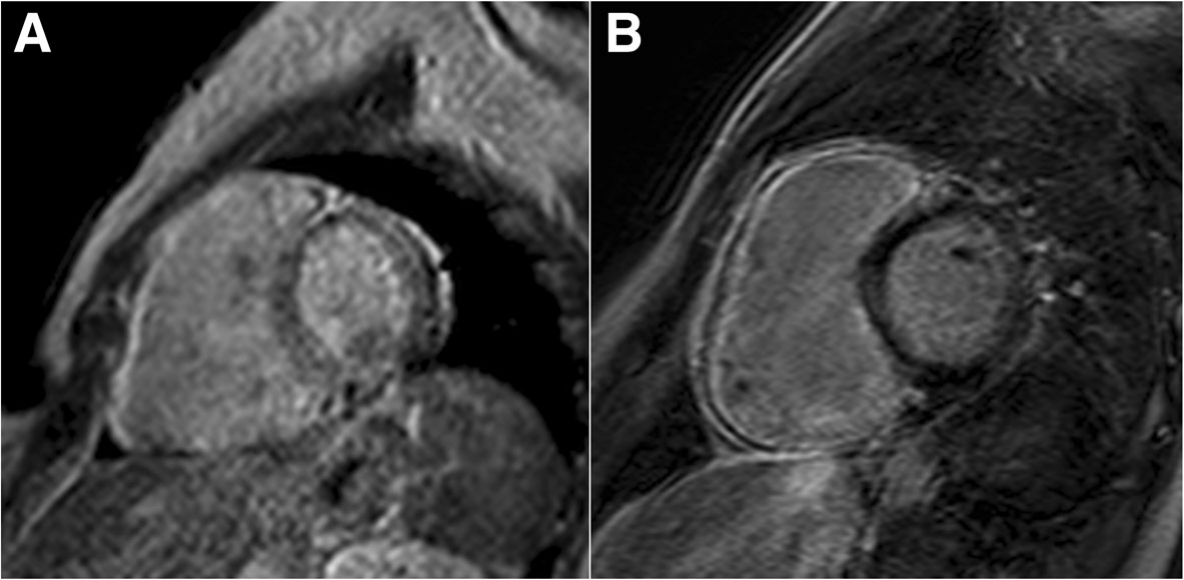
Early (**a**) and delayed (**b**) dynamic contrast enhanced 3D short axis images, showing abnormal contrast enhancement of the dilated right ventricular free wall

Echocardiography showed a normal geometry of the left ventricle, with a slightly reduced systolic function (EF: 51%), a normal diastolic pattern and mild tricuspid regurgitation; the right ventricle was dilated with evidence of hypokinesia of the apical segment and severely reduced systolic function (TAPSE: 16 mm), hinting the diagnosis of ARVD. CMR was performed using a superconducting system 1.5 T MR (Gyroscan Intera, Philips Medical System, Best, The Netherlands) with a maximum gradient capability of 30 MT/m and maximum slow rate of 150 MT/Ms. MR acquisition was triggered by ECG and included a T1-weighted (T1W) sequence and cine-MR images in the short-axis, and vertical long-axis and horizontal long-axis planes for displaying cardiac contraction. Short time inversion recovery (STIR) T2-weighted (T2W) sequence was obtained to suppress fatty tissue signal. Finally, three-dimensional (3D) T1-delayed enhancement sequences, were acquired at 10, 15 and 20 min, after the intravenous injection of Gd-DTPA at a dose of 0.1 mmol/kg. CMR revealed findings that were consistent with the major criteria of arrhythmogenic right ventricular dysplasia: dilated right ventricle with high end-diastolic volume (EDV) normalized for body surface area (BSA) (116 mL/m2); right ventricular dysfunction with ejection fraction (RVEF) of 18% and regional wall motion abnormalities (diffuse hypokinesia, free wall iper-trabeculation, dyskinesia and mid-RV free wall pseudo-aneurysm) (Fig. 2a and b). On CMR there was a slightly reduced global left ventricular systolic function (EF: 50%) with normal morpho-volumetry of the left ventricle. CMR also revealed the following findings that were consistent with a diagnosis of acute/subacute viral myocarditis: diffuse edema of the right ventricular free wall on STIR images (Fig. 3a and b), associated with sub-epicardial late gadolinium enhancement (Fig. 4a and b) of the same region. Overall, the patient met the criteria for both ARVD and acute/sub acute viral myocarditis. After the diagnosis the patient received an automatic implantable cardioverter defibrillator (AICD) and a beta-blocker (Sotalol) was prescribed at discharge. There was no recurrent VT up to the 4-month follow-up. At 6-month follow-up, the patient complained for palpitation and dizziness. AICD recordings demonstrated 5 sustained VT episodes with appropriate device intervention in all the cases. Transthoracic echocardiography demonstrated a preserved systolic function of the left ventricle (EF: 59%) and a dilated and hypokinetic RV, owing a severely reduced systolic function (TAPSE = 14 mm, fractional change area of 30%). Currently, the patient is on clinical follow-up with no apparent further complications.

## Discussion

ARVD is predominantly a genetically determined heart muscle disorder, pathologically characterized by fibro-fatty replacement of the right ventricular myocardium [1, 2]. This pathology predisposes to ventricular arrhythmias and sudden cardiac death and it can be divided in three progressive phases: in the early “concealed phase” patients are often asymptomatic; in the overt “electrical phase” patients present with symptomatic arrhythmias; finally, in the diffuse disease patients may present with right, left or biventricular heart failure with associated ventricular arrhythmias. Current Task Force Criteria for ARVD diagnosis include, repolarization or depolarization abnormalities on electrocardiography (ECG), presence of ventricular arrhythmias, morphological and functional changes, in addition to histopathology, family history, and genetic findings [2]. Our patient fulfilled the major criteria set by the Task Force [2, 3]: repolarization abnormalities (inverted T waves in right precordial leads in the absence of complete right bundle-branch block), sustained ventricular tachycardia of left bundle-branch morphology with superior axis, RV EDV to BSA ≥ 100 mL/m^2^ and RVEF ≤ 40%, as assessed by echocardiography and CMR. In addition, the patient fulfilled two of the Lake-Louise criteria for CMR in acute myocarditis [4]. Infact, CMR showed the presence of myocardial edema on STIR-T2W sequences and sub-epicardial necrosis on post-contrast T1W sequences; therefore, these imaging findings added to troponin I and CK-MB release and an elevation of CRP and white blood cells, strongly suggested an acute myocarditis. Several studies have demonstrated that myocarditis may affect the RV, causing structural abnormalities, including microaneurysms, as well as the arrhythmic manifestations that are typical of ARVD. Some cardiotropic viruses (enterovirus or adenovirus), detected in patients with sporadic ARVD, were considered to have a potential role for the disease; in fact the activation of inflammatory mediators, determined by the myocardial infection, could damage the cardiac adherents junctions and the T-tubule system, resulting in ventricular irritability and/or ion channel disruption and consequent ARVD symptomatology [5]. However, this hypothesis is controversial, because the inflammation could be a secondary event; infact, an already diseased myocardium could have an increased susceptibility to viral infection with resulting myocardial inflammation [6]. Therefore, the precise relationship between ARVD and acute myocarditis is not yet determined [7].

In a previous manuscript, Mavrogeni et al. described the case of a patient with Naxos disease, a recessively inherited arrhythmogenic cardiomyopathy (ACM), characterized by woolly hair and palmo-plantar keratoderma [8]. This patient was asymptomatic and presented with chest pain and increased troponin I. A deletion in plakoglobin (cell adhesion protein) gene (Pk2157del2TG) was identified as the cause of Naxos disease [9]; therefore, it has been proposed that a defect in proteins involved in cell–cell adhesion, particularly under mechanical stress conditions, could result in cell death and progressive myocardial fibro-fatty replacement [10]. Moreover, myocardial inflammatory infiltrates, frequently detected in patients with ARVD, have been assumed as a reaction to cell death rather than a consequence of an infective process [11]. As described in recent papers, the reactive nature of myocarditis in ARVD has been supported by the massive inflammatory cell infiltrates found after acute myocardial necrosis in early stages of disease in desmoglein-2 transgenic animal model [12]. In particular, Kant et al. have shown the etiologic role of a deletion of the adhesive extracellular domain of the desmosomal cadherin in mice with an arrhythmogenic right ventricular cardiomyopathy like phenotype, with ventricular dilation and fibrosis. Disturbed sarcomer structure, multiple autophagic vacuoles and swollen mitochondria are typical findings of this cardiomyopathy, inducing cardiomyocyte death, aseptic inflammation and fibrotic replacement, with consequent impairment of the cardiac function [13].

It must be underlined that in our case the patient started to suffer of palpitations and fatigue for light efforts after giving birth her third child in 2009. Therefore, it is reasonable to raise the hypothesis that pregnancy, the hormonal changes and/or inflammatory stress occurring during pregnancy may unmask ARVD. Due to the rarity of cases, the relation between the two events is not yet established. Furthermore, idiopathic right ventricular outflow tract ventricular tachycardia, right ventricular myocardial infarction, dilated cardiomyopathy, myocarditis, pulmonary hypertension, atrial and ventricular septal defects are possible differential diagnoses including a dilated right ventricle. Moreover, sarcoidosis with cardiac involvement can mimic ARVC, making the differential diagnosis very challenging. Uhl’s disease, an unusual cardiac disorder with almost complete absence of right ventricular myocardium, should also be considered as differential diagnosis [14]. ARVD and myocarditis have often different prognosis and treatment; in fact, the former is a complex and progressive entity that requires major clinical intervention, as AICD implantation, or heart transplantation, while the latter is often a self-limited pathology responding to a conservative approach.

To the best of our knowledge, only one case has been published in the English literature showing in a 23 years old man this rare linkage between ARVD and acute myocarditis, based on echocardiography and CMR findings [15]. In agreement with this article, in our case and according to the current international task force consensus statement for ARVD [16] the patient underwent an AICD implantation and is currently in clinical follow up, with no further complications.

In the case presented, CMR allowed the correct identification of the two different clinical entities; in addition, the enhancement pattern as shown on delayed CMR images, may also serve as a map for the exact location of ablation or an endomyocardial biopsy [17].

## Conclusions

This case shows the rare association between acute myocarditis and ARVD, highlighting the importance of a complete RV assessment through a CMR study in the context of ventricular arrhythmia. CMR is the most accurate and reproducible technique for assessing the morphology and segmental wall motion of the RV; in addition, it can evaluate the presence of myocardial inflammation with specific STIR-T2W sequences and myocardial fibrosis or necrosis with post-contrast T1W sequences, leading the physician to the correct final diagnosis and to the most appropriate management of patients presenting with ventricular arrhythmias.

## Abbreviations

3D: Three-dimensional
ACM: Arrhythmogenic cardiomyopathy
AICD: Automatic implantable cardioverter defibrillator
ARVD: Arrhythmogenic right ventricular dysplasia
BSA: Body surface area
CCU: Coronary care unit
CMR: Cardiac MR
CRP: C-reactive protein
ECG: Electrocardiography
EDV: End-diastolic volume
RV: Right ventricle
RVEF: Right ventricular ejection fraction
STIR: Short time inversion recovery
T1W: T1-weighted
T2W: T2-weighted
VT: Ventricular tachycardia
